# Re-caching by Western Scrub-Jays (*Aphelocoma californica*) Cannot Be Attributed to Stress

**DOI:** 10.1371/journal.pone.0052936

**Published:** 2013-01-09

**Authors:** James M. Thom, Nicola S. Clayton

**Affiliations:** University of Cambridge, Department of Psychology, Cambridge, United Kingdom; Utrecht University, The Netherlands

## Abstract

Western scrub-jays (*Aphelocoma californica*) live double lives, storing food for the future while raiding the stores of other birds. One tactic scrub-jays employ to protect stores is “re-caching”—relocating caches out of sight of would-be thieves. Recent computational modelling work suggests that re-caching might be mediated not by complex cognition, but by a combination of memory failure and stress. The “Stress Model” asserts that re-caching is a manifestation of a general drive to cache, rather than a desire to protect existing stores. Here, we present evidence strongly contradicting the central assumption of these models: that stress drives caching, irrespective of social context. In Experiment (i), we replicate the finding that scrub-jays preferentially relocate food they were watched hiding. In Experiment (ii) we find no evidence that stress increases caching. In light of our results, we argue that the Stress Model cannot account for scrub-jay re-caching.

## Introduction

Western scrub-jays (*Aphelocoma californica*) cache food for future times of paucity, and exhibit an array of strategies to protect their stores from theft by conspecifics [1]. Scrub-jays rely on spatial memory to locate their caches [2], and similarly, potential thieves use *observational* spatial memory to locate the food they witnessed being cached [3]. Storers will therefore sometimes wait until an observer has left to retrieve all caches and store them safely elsewhere. Scrub-jays ‘re-cache’ according to who was watching when [4], and appear to project their own experience as a thief to observers [5]. Experience projection is one of a suite of impressive intellectual abilities that have led some to describe scrub-jays and other corvids as “Feathered Apes” [6].

A recent model of re-caching presents the possibility that scrub-jays might not be directly sensitive to prior observation, but to stress [7]. In a paper titled “*Corvid Re-Caching without Theory of Mind': a Model*”, the authors argue that the motivation to re-cache is indistinct from the drive to cache, and that caching is enhanced by stress. Two main causes of stress are posited: observation by a dominant conspecific, and the belief that caches have been stolen. In the model, the perception of cache-theft results from failures of spatial memory associated with caching while observed [8]. Here, we test the hypothesis that stress, induced by *actual* cache-theft, increases caching in Western scrub-jays.

This study has two parts: First, we seek to replicate the finding that scrub-jays re-cache more after caching in front of a conspecific than they do after caching in private. Second, we assess the model's assumption that re-caching is not motivated by a desire for cache security, but by a general desire to cache more. To do so, we ask whether the same scrub-jays cache more upon discovering that earlier caches have been stolen. If they did, this would provide support for a critical prediction of the Stress Model: that *any* cause of stress can drive re-caching, irrespective of social context.

## Materials and Methods

### Subjects

Eight (4 male) sexually mature, hand-raised Western scrub-jays (*Aphelocoma californica*) participated in this study; all the birds completed both experiments. Birds were maintained at 21±1°C, on a 12∶12 hour light-dark schedule. Subjects were pair-housed in 3–4 m^3^ home cages (dimensions: 3–4 m long×1 m high×1 m deep), which could be divided into 1 m^3^ sections (1 m×1 m×1 m). Birds could be isolated by inserting dividers between the sections. Dividers were either opaque (white, plastic) or transparent (wire mesh). Home cages were visually occluded from one another, so that each bird could only see its cage partner. Subjects always had *ad libitum* access to water, and were fed on a maintenance diet of fruit, vegetables, mixed nuts, grains and seeds, bread, dog biscuits, and cuttlefish bone.

The birds cached in ice-cube trays with a 2×7 formation of potential caching sites - individual cube moulds filled with corncob caching substrate. Formations of Lego® Duplo® blocks around one or two sides of the wooden base were added to aid discrimination between caching trays. In Experiment (i), the cacheable items were skinned peanut halves that had been marked with a pattern of dots made by a cocktail stick.

### Experimental procedure

#### (i) Re-caching

Eight scrub-jays were given two consecutive chances to store peanut halves in a single caching tray. The storer was observed caching in one session, and cached in private in the other. Each peanut half was marked to indicate in which session it was cached. Later the same day, the birds were given 10 minutes to re-cache from the old tray to a new tray. We compared the number of peanut halves retrieved for re-caching from the *observed* and *private* caching sessions.

Each bird completed two trials, one as the storer, and one as the observer. Storer-observer pairs were housed together, so half the birds were storers first, and the rest were observers first. Maintenance diet was removed from the cages 2 hours prior to testing in order to motivate caching. Storers were isolated into 1 m^3^ sections using transparent dividers at one end of the home cage 15 minutes before caching began.

*Stage 1: caching.* Two 15-minute caching sessions separated by a 5-minute interval. For each session, subjects were presented with 10 peanut halves and the same single caching tray. All bowls and uncached peanut halves were removed from the cage after each session, though the tray remained in place. In one of the sessions, the storer and observer could see one another (*observed*), while in the other, they were isolated by opaque dividers and could not (*private*). Opaque dividers were inserted 5 minutes before caching in *private*. Condition order was counterbalanced between birds. In *observed* sessions, cacheable peanut halves were marked with an “X” pattern of dots, while in *private* sessions, they were marked with a line of dots.

*Stage 2: re-caching.* Later, the storers re-cached for 10 minutes *in private*. A new tray was placed in the centre of the cage floor, facing the old tray. All items retrieved for re-caching were recorded. A peanut half was retrieved for re-caching if: a) it was found in the new tray following re-caching, or b) it was removed by the storer from the tray after the caching session, and found in the cage. Those items found in the new tray or found cached within the cage (i.e. not on the cage floor) were noted as ‘re-cached’.

#### (ii) Does stress lead to caching?

Subjects were given two caching sessions followed by a single recovery period each trial. The first session was a ‘*sham*’ session, in which no food was available for caching. In the second, ‘*pilfer*’ session, subjects could cache up to 20 peanuts, but all caches were removed from the tray (out of sight of the subject) before recovery. At recovery, the birds were presented with either the *sham* or *pilfer* tray, along with a new tray, and 40 peanuts to cache. All caching/recovery sessions were conducted in private. Each subject completed two trials, one in which they recovered from the *sham* tray, and the other from the *pilfer* tray. We compared the number of items cached at recovery between the *sham* and *pilfer* conditions.

*Stage 1: ‘sham’ caching.* Subjects were food-deprived for 2 hours before testing, and isolated 30 minutes before testing. During the 15-minute *sham* caching session, subjects were presented with an empty bowl and a single tray. ‘Sham’ tray location (near/far from cage partner) was counterbalanced between birds. After *sham* caching, the bowl and tray were removed from the cages.

*Stage 2: ‘pilfer’ caching.* Fifteen minutes after *sham* caching, the birds were given a real caching session, during which they were given 20 peanuts to store in a different tray, opposite the location of the *sham* tray. After caching, the bowl, tray and any peanuts were removed from the cages. The trays were taken to a different room, and emptied of their caches and re-filled with corncob substrate.

*Stage 3: recovery.* Thirty-five minutes after the *pilfer* session, a 10-minute recovery session began. The birds were given 40 peanuts, a new tray along the centre of the front wall, and either the *sham* tray or the *pilfer* tray. The tray received for recovery on trial 1 was counterbalanced between birds. The number of caches in each tray after recovery was recorded.

### Analysis

All effects were assessed using paired t-tests, which were carried out using SPSS 18.0. The results of the first experiment (re-caching) were assessed using a one-tailed analysis because it was a replication of several previous studies, giving a clear expectation of directionality [4], [5], [9].

### Ethics statement

Work was conducted under UK Home Office project licence PPL 80/2519.

## Results

### Re-caching

**Figure 1:**
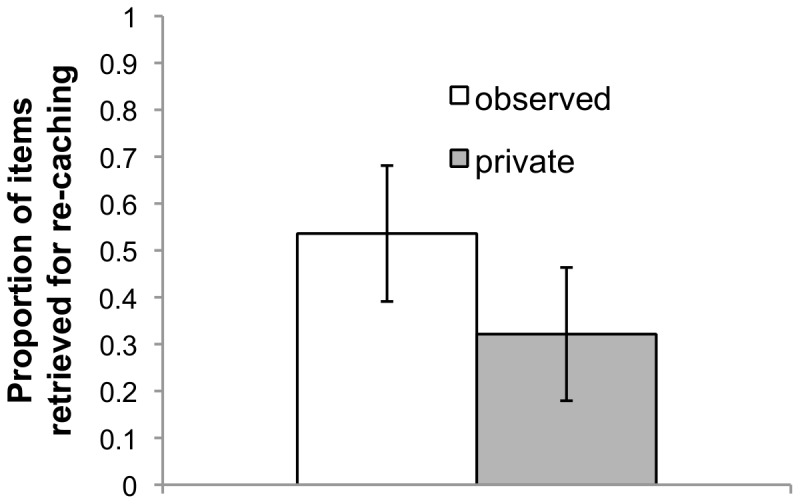
Proportions of items retrieved for re-caching in Experiment (i). Items were cached while either observed, or in private. Error bars display standard errors for each condition.

**Figure 2:**
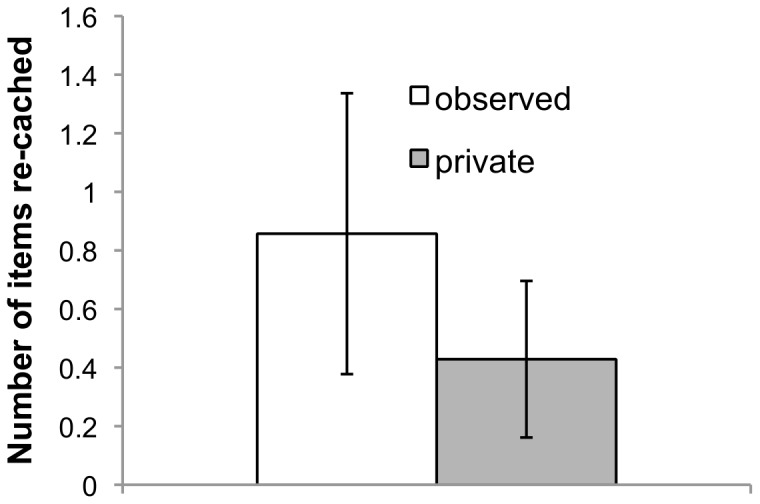
Absolute number of items re-cached in Experiment (i). Items were cached while either observed, or in private. Error bars display standard errors for each condition.

The birds retrieved for re-caching a higher proportion of peanut halves that they stored while observed, than of those stored while in private (t(7) = 2.22, p = .031, 1-tailed; Figure 1, Table S1). Of the retrieved items, more were re-cached if they were stored while observed than in private (t(7) = 2.97, p = .010, 1-tailed; Figure 2; Table S1). This study therefore replicates the finding that Western scrub-jays are sensitive to the past presence of potential thieves when re-caching.

The Stress Model further predicts increased caching while observed. However, there was no significant difference in the number of items remaining in the cage, un-cached, at the end of Stage 1 caching between the observed and private sessions (t(7) = .47, p = .654, 2-tailed).

### Does stress lead to caching?

**Figure 3:**
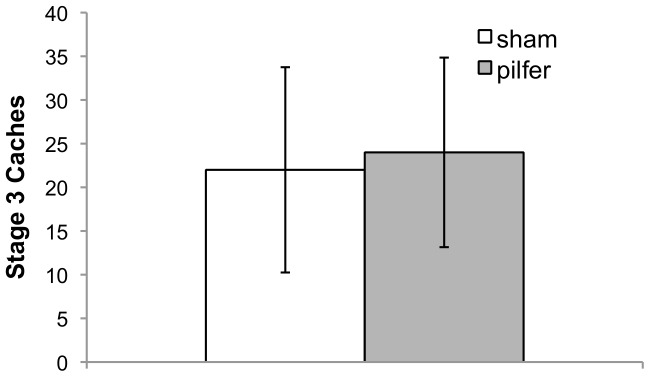
Mean number of caches made in Stage 3 of Experiment (ii) in the *sham* and *pilfer* conditions. Error bars display 95% confidence intervals.

There was no significant difference in Stage 3 caching between the *sham* and *pilfer* conditions (t(7) = −.36, p = .730, 2-tailed; Table S2), and there was almost complete overlap in the 95% confidence intervals for the two population means (Figure 3), suggesting little, if any, effect of condition on caching. In no trial did any bird cache all 40 items. Two birds cached more than 35 items in both conditions, but their exclusion did not affect the overall result (t(5) = −.32, p = .762, 2-tailed).

During Stage 2, subjects cached on average 13.69 (*sd = 5.*18) items in the *pilfer* condition, and no bird ever cached fewer than 6 items. A substantial number of stores were therefore always stolen from the pilfer tray between stages 2 and 3. In Stage 3, the birds always disturbed the substrate in empty cells, indicating they had searched for food. However, recovery failure was only surprising for the *pilfer* tray, where it was indicative of cache-theft. Subjects responded accordingly, caching a lower proportion of items in the *pilfer* tray than in the *sham* tray (t(7) = 3.8, p = .007, 2-tailed; Table S2). In summary, the scrub-jays did appear sensitive to loss of caches they had made in Stage 2, but this did not drive caching in Stage 3.

## Discussion

We have shown that Western scrub-jays do not cache more in response to the stress of cache-loss, but do avoid previously pilfered sites [10]. We also replicated the finding [4], [5], [9] that scrub-jays re-cache more following a caching session in which they were observed than after caching in private. Taken together, these results critically undermine the claim that re-caching is driven by stress.

The Stress Model does not distinguish between caching and re-caching, modelling both as the behavioural output of a general drive to cache more. According to the model, re-caching takes place at the initial caching session as well as the later re-caching session, in both cases driven by a behavioural state called stress. The central assumption of the Stress Model is that caching while observed is associated with making more memory errors at retrieval, which elicits more stress, which drives more caching/re-caching. Confabulations would cause stress due to the surprise of not discovering caches where they were expected.

According to the model, the increase in memory errors at retrieval results from increased caching/re-caching during the initial caching session. Despite this, the scrub-jays did not seem to cache more items when observed in the first experiment. However, we only measured the total number of items cached, not the number of times each item was cached. We cannot rule out the possibility that a stress-induced increase in the drive to cache manifested itself in enhanced re-caching only.

More conclusively, we found no evidence that the surprise of cache-loss motivates caching. Compared with a control condition in which the birds would be unsurprised to find no stores, caching did not increase. The similarity in caching behaviour between the two conditions is marked; the effect of complete cache theft ought to be far stronger than the confabulation-induced perceived theft that is supposed to drive re-caching behaviour.

Further, the contrasting and clear effect of prior observation on re-caching in the first experiment presented is not easily accounted for by the Stress Model. Unlike previous studies, the same caching tray was used for both the observed and private sessions. According to the model, a stress-inducing failure to retrieve an item ought to drive re-caching of nearby items. There is nothing in the model to suggest that the birds should selectively re-cache items they cached while observed, ignoring equally near items that had been cached in private. This behaviour suggests a specific sensitivity to the social context in which the item was stored.

Van der Vaart and colleagues lay out 4 clear and testable predictions of the Stress Model. First, the birds should re-cache more *during the initial caching session* if they were observed. Second, that re-caching in the second session should only start after several surprising recovery failures. Third, emptier trays should result in more recovery failures and therefore more re-caching. Finally, the authors predict that *any* cause of stress should elicit enhanced re-caching, or caching. We assess this final prediction. We find no evidence that surprising recovery failures result in more caching.

Our study differs from the standard re-caching paradigm only in two respects. First, we increase recovery failure in the pilfer condition by stealing the birds' caches. This should elicit stress, according to the model. Second, at recovery, the birds can cache new items rather than re-cache old ones; the Stress Model explicitly equates caching and re-caching. Taken together, our results represent a strong argument against this central plank of the Stress Model of re-caching.

A major claim of the Stress Model is its simplicity. Competing arguments resort to complex cognition [11], [12] or to a plethora of ad-hoc behavioural rules based on hypothetical life experience, each tailored to a specific study. The results presented here challenge this simplicity, suggesting that caching and re-caching are, at least partially, governed by different drives.

Taking the results of this study together with existing literature, we argue that re-caching is directly sensitive to the social context in which the stores were originally made. This study was designed to test one of four central predictions of a computational model of corvid re-caching. Modelling of this kind promises to be a powerful tool for generating testable hypotheses. We would welcome further computational work focusing on the mechanisms of caching in a social setting.

## Supporting Information

Table S1 Per-bird results of Experiment (i): scrub-jay re-caching of items that were stored either in private or while observed by a conspecific.
(DOC)

Table S2 Per-bird results of Experiment (ii): scrub-jay Stage 3 caching.
(DOC)
