# Supplementary material for: Re-caching by Western Scrub-Jays (Aphelocoma californica) Cannot Be Attributed to Stress
Source: PLoS One. 2013 Jan 9;8(1):e52936. doi: 10.1371/journal.pone.0052936 (PMC3541402; doi:10.1371/journal.pone.0052936)
Supplement: Table S1 — Per-bird results of Experiment (i): scrub-jay re-caching of items that were stored either in private or while observed by a conspecific. (DOC) [file pone.0052936.s001.doc]

|  | **Proportion retrieved for re-caching** | | **Number of peanut halves re-cached** | |
| --- | --- | --- | --- | --- |
| **Bird** | **Observed** | **Private** | **Observed** | **Private** |
| 224 | 0.38 | 0.10 | 3 | 1 |
| 225 | 1.00 | 0.75 | 2 | 1 |
| 217 | 0.67 | 0.00 | 0 | 0 |
| 215 | 0.80 | 1.00 | 3 | 2 |
| 222 | 2.86 | 0.00 | 2 | 0 |
| 223 | 1.00 | 0.50 | 1 | 0 |
| 39 | 0.00 | 0.00 | 0 | 0 |
| 108 | 0.00 | 0.00 | 0 | 0 |
