# Supplementary material for: Re-caching by Western Scrub-Jays (Aphelocoma californica) Cannot Be Attributed to Stress
Source: PLoS One. 2013 Jan 9;8(1):e52936. doi: 10.1371/journal.pone.0052936 (PMC3541402; doi:10.1371/journal.pone.0052936)
Supplement: Table S2 — Per-bird results of Experiment (ii): scrub-jay Stage 3 caching. (DOC) [file pone.0052936.s002.doc]

|  | **Number of Stage 3 caches** | | **Proportion of Stage 3 caches in the old tray** | |
| --- | --- | --- | --- | --- |
| **Bird** | **Sham** | **Pilfer** | **Sham** | **Pilfer** |
| 224 | 38.5 | 38 | 0.47 | 0.16 |
| 225 | 1 | 13.5 | 1.00 | 0.00 |
| 217 | 8 | 21.5 | 0.75 | 0.28 |
| 215 | 26 | 23 | 0.62 | 0.04 |
| 222 | 37 | 39 | 0.32 | 0.41 |
| 223 | 34 | 6 | 0.32 | 0.00 |
| 39 | 18 | 13 | 0.39 | 0.15 |
| 108 | 13.5 | 38 | 1.00 | 0.26 |
